# Potential of olive oil tourism in promoting local quality food products: A case study of the region of Extremadura, Spain

**DOI:** 10.1016/j.heliyon.2019.e02653

**Published:** 2019-10-21

**Authors:** José Antonio Folgado-Fernández, Ana María Campón-Cerro, José Manuel Hernández-Mogollón

**Affiliations:** a Department of Financial Economics and Accounting, University of Extremadura, 10071, Cáceres, Spain; b Department of Business Management and Sociology, University of Extremadura, 10071, Cáceres, Spain

**Keywords:** Olive oil tourism, Local food, Identity, Emotional image, Overall experience, Tourist loyalty, Tourism management, Tourism, Education, Economics, Business

## Abstract

This work aims to highlight tourism based on gastronomy, heritage and olive oil-related activities. These activities enhance the knowledge about the product itself and its links with culture and local traditions. Olive oil tourism is based on a typical gastronomic product of internationally recognised quality. This type of tourism boosts sustainable destinations capable of introducing and promoting the consumption of local products. In addition, it favours the implementation of different activities in rural settings, such as routes through the olive groves, gastronomic markets, educational initiatives, historical events, etc. This study analyses the development of olive oil tourism in the district of La Serena in Extremadura (Spain). The study involved 208 tourists that were asked about their experience on oil tourism during their trip. The results emphasize the great potential of development that olive oil tourism can achieve in rural settings as sustainable destinations. From the results obtained, managers of rural settings with a great olive oil tradition can base their tourism strategies on the gastronomic and cultural context of this local product.

## 1 Introduction

In general, the indigenous and quality agricultural foods that are produced in a territory provide remarkable economic potential for this activity. Thus, the consumption of certified quality local foods can be an important component for the economic development of the territory where they are produced (Frisvoll et al., 2016), since they are sometimes capable of combining healthy, natural, local and traditional values (Perito et al., 2009), linked to areas with great gastronomic heritage (Veeck et al., 2016).

On the other hand, it is known that industrial food has environmental, social and economic problems associated with its production. New patterns of consumption focused on local artisanal foods made by small local producers, could help the sustainability of a territory. Therefore, indigenous food can be an element to differentiate and positively value a place (Alonso and Krajsic, 2013).

Olive oil tourism, also known as *“oleotourism”*, offers activities based on the olive groves, their history, the olive and the olive oil. In this way, olive oil has generated a new offer in tourism where visitors can enjoy the landscapes of the olive groves and taste different varieties of olive oil. Spain has become the world's leading producer of both olive oil in general, and extra-virgin olive oil. It is currently recognized, both for the extension of its crops and for the high quality of the product (Torres-Ruiz et al., 2018). The region of Extremadura, located in the southwest of Spain, accommodates, in its two provinces, 135 mills producing olive oil (85 in the province of Badajoz and 50 in the province of Cáceres). These facts suggest the importance of *“oleotourism”* for a territory traditionally recognized as a destination with large natural spaces and quality local cuisine. This differentiated tourism requires identifying and knowing the travellers who practice it, in order to determine strategies for the territory that favour the sustainable development and economic progress of the local community (Millán-Vazquez de la Torre et al., 2017).

The present study analyses the potential of tourism based on olive oil, and how it can contribute to the sustainable development of the territory where it is produced. This type of tourism has become an emerging model, an alternative to traditional tourism, since *“oleotourism”* provides the traveller with a more active and participative role around a high-quality gastronomic product. Tourism based on food, heritage and activities revolving around olive oil help to improve the knowledge of the product itself and to strengthen its links with culture, local traditions and sustainability (Robinson and Getz, 2016).

The main objective of this work is to identify the areas of development of *“oleotourism”* as a sustainable alternative of the territory, based on the profile of these tourists. To this end, the study evaluates the possibilities of tourism through the analysis of data derived from a survey of olive oil producers in the Extremadura region. The present study, therefore, focused the secondary objectives on analysing this type of tourism, the different variables that integrate it and the agents involved in its development.

## 2 Theory

### 2.1 Local food, tourism and identity of the territory

The local economy can be benefited through the development and commercialization of quality local foods that are produced in it. The territory can grow economically, the industry can create jobs, reinvest profits and contribute to the payment of local taxes for the community. Both the agricultural sector and tourism can create an identity for the destination based on culture and history in the place where a unique recognized food is produced (Henderson, 2009).

The features of the identity of a place are shaped by a set of beliefs, values and images that the tourist specially and unequivocally associates to a place (Dredge and Jenkins, 2003). This identity can be based on unique values such as its environment, its natural, historical and cultural heritage, or its gastronomy (Antunes, 2000).

Traditional agriculture can preserve the specific way of life of different geographical areas, centred on the rural world, on gastronomy, cultural roots and traditions (Mitchell and Hall, 2006). As such, local markets give farmers the opportunity to diversify and promote their food to a wider public through tourism. For example, farmers' markets with local products are increasingly becoming a focus of attraction not only for the traditional local consumer, but also for tourists eager to known these types of products (Hall and Gössling, 2013). Agrotourism is related to the promotion of quality, respect for the local heritage and environment, and local character, although their practices may vary from territory to territory (Santa Cruz et al., 2019).

This trend is explained by the growing value of local products of recognized quality, such as organic foods like extra virgin olive oil (EVOO), since they have become a genre that fulfils other factors beyond their function to satisfy the basic need for food. They can be an experience and an opportunity for education and entertainment (Perito et al., 2009). Consequently, the identity and territorial links of the product with the territory, provide a greater understanding of the contribution to the sustainability of a territory through gastronomy and tourism (Everett and Aitchison, 2008).

To promote the sustainability and identity of a territory combined with tourism development, it is convenient to analyse tourism offer and demand, and its impact. Thus, in the case of *“oleotourism”,* the olive groves provide a great landscape and environmental culture, where tourist activities can contribute both to promote its conservation and to promote regional economic development through the purchase of products.

In addition, food has a social dimension that arises when its consumption is shared or the preparation of a dish is done. This new context promotes the idea that quality local foods are part of the tourism and leisure sectors (Kivela and Crotts, 2006). In this context, the predominance of traditional cuisine in rural areas is an opportunity for development, since food can be offered to tourists to meet their culinary, cultural, historical and leisure needs during their trip (Hillel et al., 2013). This approach makes quality local foods become tourist resources in themselves, contributing to the development of tourism and the promotion and commercialization of it (Armesto and Martin, 2006).

Tourist destinations with quality regional foods increasingly focus their promotion and economic development on the enhancement of their local gastronomy as an identity (Gálvez et al., 2017). Thus, for tourism to contribute to the sustainability of a destination, not only will it consist in the purchase and tasting of its local foods, it will also require that local producers to use specific smaller scale techniques or ones with less environmental impact than large industries. In this context, a change of mentality is possible where sustainability can become a successful strategy for the tourism sector (Santa Cruz et al., 2019).

As a result, this type of gastronomic tourism can consolidate the regional productive culture, while improving the economic development, identity and sustainability of the territory, since it is usually small producers that protect the environment and the local economy (Everett and Aitchison, 2008).

### 2.2 Experiences and olive oil tourism

A tourist destination is more than a mixture of natural, cultural and artistic resources. It becomes clear that there is an ever increasing need to guarantee the experience that the tourist is looking for when visiting the destination (Cracolici and Nijkamp, 2008). In the case of *“oleotourism”,* tourist products based on experiences on olive oil are offered. Its enjoyment revolves around the world of sensations that the visitor perceives through their senses and emotions (Torres-Ruiz et al., 2018).

Thus, olive oil tourism is based on the set of activities created around olive oil. These activities may include visits to olive groves, historic and current mills (sometimes coinciding with the harvest of olives), routes (similar to those that have already been developed in the world of wine) and landscapes, oil tasting or tasting typical local dishes, in which olive oil is the star ingredient. So, “*oleotourism*” can include multiple cultural activities related to nature, local heritage, the environment and the culture of the territory (Millán-Vazquez de la Torre et al., 2017).

Olive oil routes are one of the most important products of olive oil tourism. These routes are defined as an itinerary that allows farmers, small producers and hoteliers to explain the production process of olive oil and its presence in regional cuisine. This cuisine offers traditional dishes based on the local primary produce of the area, which is considered an expression of the cultural identity of the territory (Schlüter and Ellul, 2008). As part of this cultural heritage, tourists can also take advantage of itineraries and tourist activities (Riley, 2005) as a means to learn how the product is transformed from the olive grove to become olive oil (Torres-Ruiz et al., 2018).

Food-based tourism is currently one of the best means to promote unique experiences in tourist destinations, due to the increasing importance that tourists attribute to knowledge of the territory's gastronomy. For these tourists, local dishes may be one of the reasons that people choose to visit a place. For example, to try a typical dish or learn more about cooking in a certain area (Tsai and Wang, 2017).

Tourism based on quality indigenous foods offers multiple proposals for travellers (Shaw, and Williams, 2004). Very often they consist of activities and experiences that cost more than other tourism modalities. Several studies suggest that there is a greater interest in gastronomic tourism for those tourists with greater purchasing power. These tourists are willing to spend more on their trip in exchange for being able to enjoy an authentic experience based on quality food from producers (Everett and Aitchison, 2008). In addition, through the experience lived at the destination, tourists can perceive an affective image of the territory (Baloglu and Brinberg (1997), which can be improved with the enjoyment of agri-food products and local cuisine (Peštek and Činjarević, 2014).

Sustainable tourism is practiced by those tourists that appreciate the preservation of the culture, society, environment and economy that is based on the local products of the territory they visit (Dinan and Sargeant, 2000; Lee, 2013). In this way, this tourist respects and values the local culture linked to the natural environment of the destination they visits (Mehmetoglu, 2010).

Finally, positive experiences in a sustainable destination where quality local food is produced can positively influence the tourist's loyalty. The possibility of revisiting the same destination in the future, or recommending food-based experiences, is associated with a positive overall assessment of the experience perceived by tourists at the destination (Hui et al., 2007). Thus, these experiences may have a greater role for the future intentions of the tourist, compared to other travel or tourist attractions (Kozak and Rimmington, 2000). In addition, the combination of quality local products in rural settings can further enhance the tourist's intentions when returning to the destination in the future or recommending their experience to their friends and relatives (Vujko et al., 2017).

**Fig. 1:**
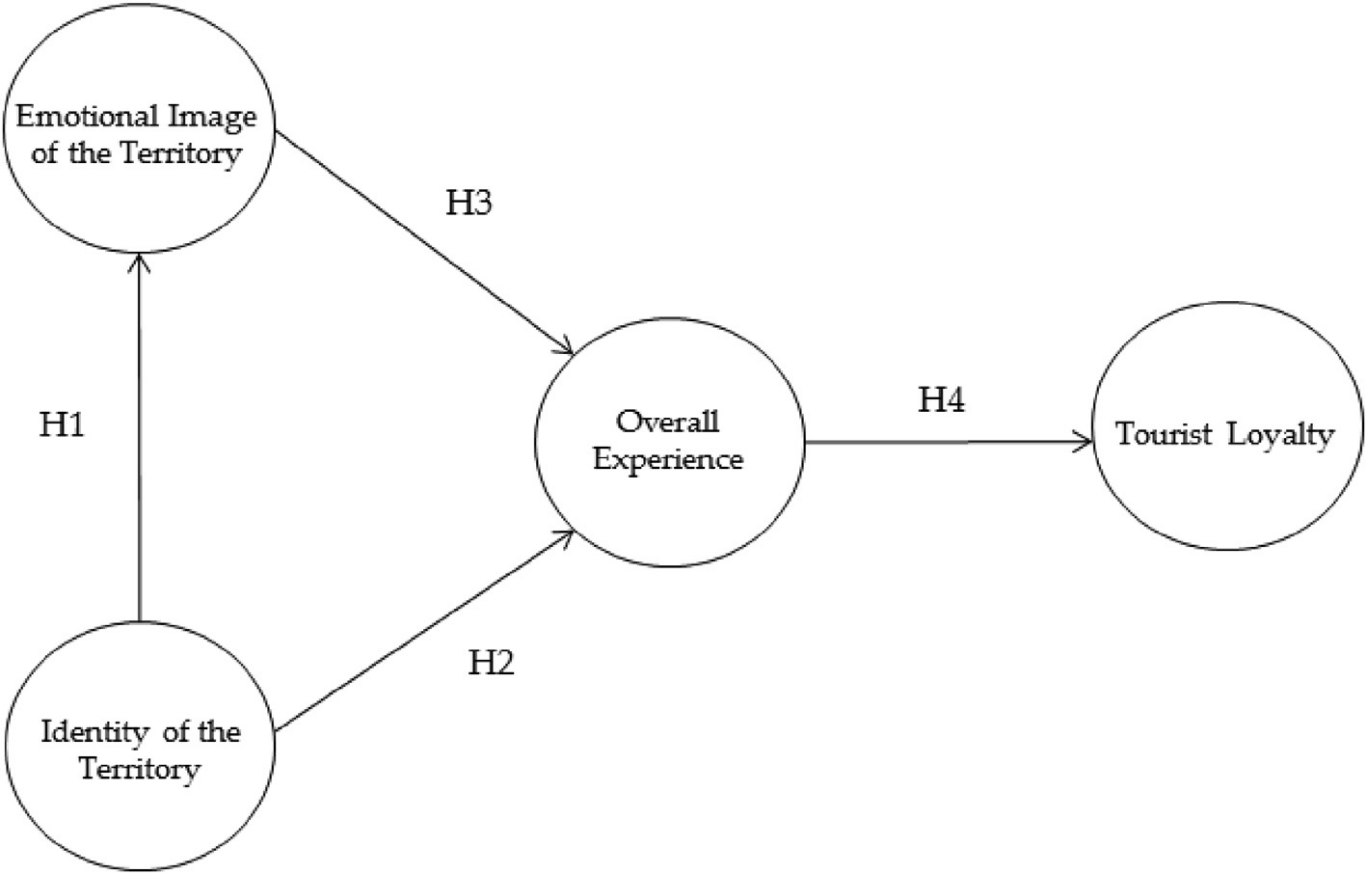
Proposed model.

All this is specified in the following hypotheses that can be observed in Fig. 1. Thus, the figure contains the theoretical model proposed to be tested with the empirical study, in which the relationships between variables are connected as research hypotheses.

H1 There is a positive relationship between the identity of the place and the emotional image of the territory.

H2 There is a positive relationship between the identity of the place and the overall experience of the tourist.

H3 There is a positive relationship between the emotional image of the territory and the overall experience of the tourist

H4 There is a positive relationship between the overall experience of the tourist and the tourist loyalty.

## 3 Materials & methods

### 3.1 Description of the geographical area^1^

La Serena is located in the southwest of Spain, in the province of Badajoz (Extremadura). Badajoz is the largest Spanish province with 21,766 km^2^. Within it, the territory of La Serena bases its economy mainly on the agricultural sector, and especially on the production of EVOO. The town of Monterrubio de la Serena is the epicentre of olive oil production in the area and has the Olive Oil Museum, an emblematic building recognized as having a fundamental role to play for learning the history that unites the territory with the EVOO. It also carries out different activities related to olive oil such as tastings, sampling, workshops...etc.

Spain has 32 protected designation of origin (P.D.O) of EVOO. Of these, two correspond to the region of Extremadura: Gata-Hurdes, and Monterrubio in the territory of La Serena. The Protected Designation of Origin Monterrubio Olive Oil includes in its geographic scope some 15 localities. The product made based on this P.D.O. is a virgin olive oil obtained mainly (90%) from the ergot and *picual* olive varieties, together with a small percentage of other specific varieties of the territory.

The olive is favoured by the particular microclimate of the area, achieving a unique olive oil, with unparalleled organoleptic characteristics and with an acidity expressed in oleic acid no higher than 0.8 grade per 100 grams. The olive oil produced in this area is yellow-green, with a fruity, aromatic, slightly spicy and almond flavour. To ensure the highest quality of the product and the sustainability of the territory, only olives harvested directly from the tree are used. In this way an oil of extraordinary quality recognized internationally is obtained.

In this way, the territory of La Serena is gradually becoming a meeting point for tourists who come to the area in search of its rich cultural heritage and quality food. Its gastronomy is based on the local products that are produced in that territory, mainly those foods derived from the Iberian pig and organic farming. Among the latter, olive oil stands out as one of the most important of the primary products produced locally, and is present in most of the typical dishes of the territory.

### 3.2 Questionnaire and scales

A quantitative approach of research was implemented. A personal survey ‘face to face’ was conducted with the assistance and cooperation of three trained people for conducting the fieldwork professionally, using a structured questionnaire hosted in a website, what favours data collection and avoids any error of transcription of the answers in the statistical software, since the online platform registers data automatically. The language used in the questionnaire was Spanish, but with foreigners with difficulties in understanding Spanish, English was used. It only affected a very reduced group of tourists questioned.

The fieldwork was done during weekends from the 15^th^ to the 31^st^ of March 2019 in the territory of La Serena (Extremadura, region of Spain). It was mainly done in the town of Monterrubio de la Serena, it having the Olive Oil Museum, it attracts many visitors and can be considered the epicentre of this territory in terms of olive oil. Potential respondents were intercepted and asked for the voluntary participation in the survey. Confidentiality was also ensured. Interviewers read each question and possible answers to each enquired tourist, and simultaneously transcripted the response into the online questionnaire. A total of 208 valid questionnaires were obtained using a non-probability sampling of convenience in the cited museum. We obtained a sufficient sample, according to the requirements of the use of PLS (Hair et al., 2000; Asmelash and Kumar, 2019).

The questionnaire was divided into four sections. The blocks refer to the opinion that tourists have about the emotional image of the destination, the identity of the territory, their general experience as a tourist and the loyalty in terms of their willingness to return or recommend the visit to the destination and/or activities based on olive oil. This part consisted of questions evaluating the different variables included in the proposed model. To measure each variable, adapted scales were used, already contrasted in previous studies (Hui et al., 2007; Kim et al., 2012; López-Guzmán and Sánchez-Cañizares, 2012) as shown in Table 1. The questionnaire closes with some questions about the motivations and the demographic profile of tourists. Variables were measured on a five-point Likert scale (1: totally disagree, 5: totally agree).

**Table 1:** Scales used.

Authors	Dimension	Indicators
Baloglu and Brinberg (1997)	Emotional image of the territory (EI)	(EI1) The territory of La Serena is nice (EI2) The territory of La Serena is entertaining (EI3) The territory of La Serena is relaxing (EI4) The territory of La Serena is exciting



Filo et al. (2013)	Identity of the territory (IT)	(IT1) The “oleotourism” of this territory means a lot to me (IT2) I am very attached to the EVOO of this territory (IT3) No other “oleotourism” location can be compared to the territory of La Serena (IT4) I feel involved with the EVOO of this territory (IT5) This territory of La Serena is a good place to learn about EVOO (IT6) Visiting the territory of La Serena broadens my knowledge about EVOO (IT7) I will not forget my experience in the territory of La Serena
Kao et al. (2008)		

Cole and Scott (2004)		
Quadri-Felitti and Fiore (2013)		
Stern and Krakover (1993) Back and Parks (2003) Oh (2000)	Overall experience (OE)	(OE1) The overall experience in this territory is positive (OE2) It has been worth coming to the territory of La Serena (OE3) The territory of La Serena is a good place to visit (OE4) The territory of La Serena has a good reputation
	Tourist loyalty (TL)	(TL1) I will return to the territory of La Serena (TL2) I will recommend visiting the territory of La Serena (TL3) I will return to activities related to EVOO (TL4) I will recommend the activities related to the EVOO
Chi and Qu (2008)		

The first draft of the questionnaire was revised by a panel of seven experts and professionals on tourism marketing and management. The questionnaire was revised and improved according to the suggestions received.

The IBM SPSS Statistics Version 21 program was used to carry out the descriptive analysis of the data. The Smart PLS 3.2.7 program was used to examine structural equations (SEM). The SEM method was considered the most appropriate to validate the hypotheses and confirm the models of complex relationships (Richter et al., 2016).

### 3.3 Ethical concerns

The authors' institution did not require any ethical approval for this study as no detailed, nor individual information is included. Data collected can not be linked with a specific respondent and have always been treated in a aggregate manner. Anonymity is guaranteed throughout the research.

## 4 Results

The main findings from the field work are detailed below. They are presented in blocks, in a differentiated manner for the different topics addressed. Thus, the main sociodemographic characteristics of the tourists surveyed for this study who visit the territory of La Serena are shown in Table 2. The data show a uniform distribution between both genders, although with a slightly higher percentage of men (Male: 53.4%; Female: 46.6%). As for the age groups, a clear concentration is found in the group between 40-59 years of age, which brings together more than half of the tourists surveyed (52.4%). There is also a high percentage of tourists (65.4%) with university studies within the group of the sample. This data is in agreement with other works that present the gastronomic tourist as a visitor of higher academic qualifications when compared to other types of tourists (Everett and Aitchison, 2008). The majority of tourists surveyed were Spanish (90.7%). Among the non-Spaniards, the countries of Portugal, Germany, the United Kingdom, France, Italy and the United States are represented.

**Table 2:** Socio-demographic profile of the sample group.

Variable	Sample group (%)
*Sex*	
Male	53.4
Female	46.6
*Age*	
Under 25	2.9
26–39	26
40–59	52.4
Over 59	18.8
*Level Education*	
Basic studies	1.4
High school education	20.2
Vocational training	8.2
University studies	65.4
Other studies	4.8

Regarding the main reasons that motivated the tourist to visit the destination, the different categories were analysed according to the value of their means ($\bar{\text{X}}$). As can be seen in Table 3, where absolute figures, the means and standard deviation are presented, the local cuisine ($\bar{\text{X}}$ = 4.79) represents the fundamental aspect in the tourist's decision to visit the destination. Then, with practically the same results, they learn from the EVOO culture ($\bar{\text{X}}$ = 4.64) and travel with family and friends ($\bar{\text{X}}$ = 4.63). In fourth and fifth positions are the historical-cultural heritage ($\bar{\text{X}}$ = 4.56) and rest and relax ($\bar{\text{X}}$ = 4.54). The other categories and their means are to participate in the olive oil museum activities ($\bar{\text{X}}$ = 4,13), enjoy nature ($\bar{\text{X}}$ = 4,29), walk the Camino de Santiago ($\bar{\text{X}}$ = 2,60), visit friends and relatives ($\bar{\text{X}}$ = 2.41) and for work ($\bar{\text{X}}$ = 1.89).

**Table 3:** Motivations.

Motivations	1	2	3	4	5	Min.	Max.	$\bar{\text{X}}$	σ
(M1) Activities Olive Oil Museum	0	1	29	120	58	2	5	4.13	0.650
(M2) Visit family and friends	9	130	46	21	2	1	5	2.41	0.769
(M3) Enjoy nature	0	1	12	120	75	2	5	4.29	0.594
(M4) Historical and cultural heritage	0	0	0	92	116	4	5	4.56	0.498
(M5) EVOO culture	0	0	2	71	135	3	5	4.64	0.501
(M6) Local Cuisine	0	0	0	43	165	4	5	4.79	0.406
(M7) Rest and Relaxation	0	0	7	82	119	3	5	4.54	0.563
(M8) Travel with family and friends	0	2	15	42	149	2	5	4.63	0.662
(M9) For work	63	116	20	6	3	1	5	1.89	0.798
(M10) Walk the Camino de Santiago	12	108	58	11	19	1	5	2.60	1.007

In light of the results obtained, it can be said that the local cuisine and the desire to know more about the EVOO culture can be two of the best ways to promote and consolidate tourist destinations with quality local products in sustainable territories. This is due to the increasing importance that tourists attribute to knowledge of the destination's gastronomy. These tourists have as their main motivation the quality local cuisine and learning more about the typical foods of a certain area (López-Guzmán et al., 2016).

### 4.1 Analysis of measurement model

For the analysis and study, the model of Fig. 1 was proposed, which includes the hypotheses presented for this research. The evaluation of the measurement model was carried out by means of the analysis of the validity and reliability of the different constructs, differentiating those that are reflective from the formative ones. The main difference between reflective and formative constructs is that formative measures represent instances in which the indicators cause the construct, whereas reflective indicators are caused by the construct itself. Thus, an attempt was made to evaluate if the theoretical concepts are measured in a precise way by means of the observed variables (Fornell and Larcker, 1981).

The first step consisted in analysing the individual reliability of the indicators, the internal consistency of the construct, the convergent validity and the discriminant validity of the reflective constructs (global experience and tourist loyalty) (Fornell and Larcker, 1981). The results shown in Table 4 indicate an adequate individual reliability of the items, since all the loads are well above the minimum threshold required of 0.505 (Fornell and Larcker, 1981). The analysis also indicated that all charges are statistically significant at the 99.99% level.

**Table 4:** Loadings and weights.

Construct	Indicators	Weights	Loadings
Emotional image of the territory (EI)	(EI1) The territory of La Serena is nice	0,126	
	(EI2) The territory of La Serena is entertaining	0,735	
	(EI3) The territory of La Serena is relaxing	0,324	
	(EI4) The territory of La Serena is exciting	-0,326	
Identity of the territory (IT)	(IT1) “Oleotourism” in La Serena means a lot to me	0,371	
	(IT2) I am very attached to the EVOO of this territory	0,227	
	(IT3) No other “oleotourism” location can be compared to LS	0,177	
	(IT4) I feel involved with the EVOO of La Serena	0,358	
	(IT5) La Serena is a good place to learn about EVOO	0,122	
	(IT6) Visiting La Serena broadens my knowledge about EVOO	0,225	
	(IT7) I will not forget my experience in La Serena	0,056	
Overall experience(OE)	(OE1) The overall experience in La Serena is positive		0,756
	(OE2) It has been worth coming to La Serena		0,830
	(OE3) La Serena is a good place to visit		0,792
	(OE4) La Serena has a good reputation		0,744
Tourist Loyalty (TL)	(TL1) I will return to La Serena		0,766
	(TL2) I will recommend visiting La Serena		0,785
	(TL3) I will return to do activities related to EVOO		0,808
	(TL4) I will recommend the activities related to the EVOO		0,718

To determine the reliability of the scales, the values of Cronbach's alpha coefficient (0.786, 0.770) were calculated, where all the figures reached were greater than the minimum threshold of 0.6. On the other hand, the composite reliability for the two reflective constructs (0.862, 0.853) is greater than 0.7, thus validating the internal consistency of the model (Fornell and Larcker, 1981). The convergent validity was evaluated from the mean variance extracted by construct (AVE), where all the two constructs presented values (0.610, 0.593) higher than the recommended minimum of 0.50 (Hair et al., 2000; Chin, 2010) (see Table 5).

**Table 5:** Cronbach's alpha, composite reliability, AVE (Average Variance Extracted).

Dimension	Cronbach's alpha	Composite reliability	AVE^1^
Overall Experience	0,786	0,862	0,610
Tourist Fidelity	0,770	0,853	0,593

Finally, the property of discriminant validity was verified when verifying that the correlations between the constructs are less than the square root of the average extracted variance (Falk and Miller, 1992). Based on the values reached for the measurement model, it was considered valid and reliable, so it makes the following analysis of the structural model.

### 4.2 Evaluation of structural model

In this section, once the sample measurement model has been validated, the evaluation of the internal model is carried out. In this way we try to contrast the hypotheses raised between the constructs. So, the value of R^2^ and the significance of the paths are analysed (Fornell and Larcker, 1981). The explained variance of the endogenous constructs is calculated by analysing the R2, in order to analyse the predictive power of the model (Chin, 2010). As can be seen in Table 6, the model explains 32.9% of the emotional image of the territory, 35.3% of the overall experience and 42.8% of tourist loyalty. These data indicate that the model has a high predictive value and is capable of explaining endogenous constructs (Falk and Miller, 1992). Identity and emotional image have similar capacity to shape overall experience.

**Table 6:** Effects on endogenous variables.

Construct/Hypothesis	R^2^	Direct Effect (β)	Correlation	Explained Variance
*Emotional image*	0.329			32.9%
H1(+): Identity of the territory > Emotional image		0.573	0.573	
*Overall Experience*	0.353			35.3%
H2(+): Identity of the territory > Overall Experience		0.336	0.527	17.51%
H3(+): Emotional image > Overall Experience		0.333	0.526	17.70%
*Tourist loyalty*	0.428			42.8%
H4(+): Overall Experience > Tourist loyalty		0.654	0.654	

Regarding the study of the standardized path coefficients β and the selection of critical values for the Student's t distribution, the values of the β coefficients that relate the constructs of the model, reach values greater than 0.2, and present a value of t statistic greater than 1.96 (Falk and Miller, 1992). These data can be seen in Table 7 where the results for the structural model are presented. The analysis of the meaning of the paths revealed that all the research hypotheses received empirical support from the results, thus validating the proposed model.

**Table 7:** Hypothesis support.

Hypothesis	(β)^1^	t-Value^2^ Bootstrap	Support
H1(+): Identity of the territory > Emotional image	0.573***	14.35	Yes
H2(+): Identity of the territory > Overall Experience	0.336***	4.6	Yes
H3(+): Emotional image > Overall Experience	0.333***	4.1	Yes
H4(+): Overall Experience > Tourist loyalty	0.654***	13.6	Yes

^1^ Path Coefficient. ^2^ Critical t-values: *p < 0.05; **p < 0.01; ***p < 0.001; ns not significant (based on t (4999), one-tailed test); t(0.05; 4999) = 1.645; t(0.01; 4999) = 2.327; t(0.001; 4999) = 3.092.

**Fig. 2:**
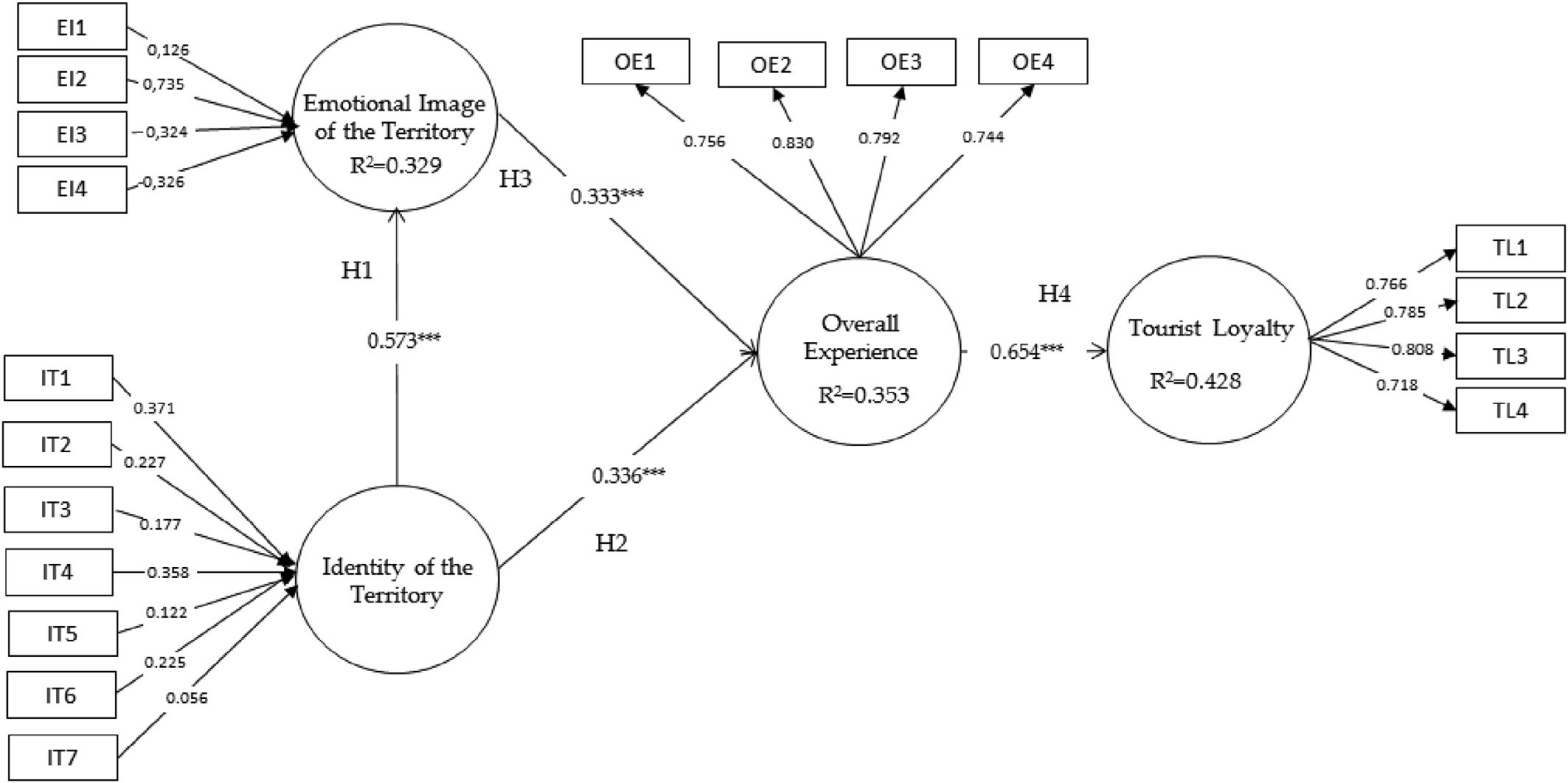
Graphical results of structural model assessment. Critical t-values: *p < 0.05; **p < 0.01; ***p < 0.001; ns not significant (based on t(4999), one-tailed test); t(0.05; 4999) = 1.645; t(0.01; 4999) = 2.327; t(0.001; 4999) = 3.092.

The graphic summary of the evaluation of the measurement and structural model can be seen in Fig. 2.

## 5 Discussion

The results obtained with the evaluation of the proposed model are in line with the findings of other previous studies. The impacts of the identity of the destination in the emotional image of the same (H1) agree with Pike and Ryan (2004) and the findings of Baloglu and Brinberg (1997). Consequently, the positive impact on the emotional perceptions of tourists suggests that tourists are strongly linked to the emotional part of the territory based on its identity. Regarding H2, the results are in line with those of Llodrà-Riera et al. (2015), confirming that the identity of the territory influences the overall tourist experience. According to these authors, this is a clear result, since the attractions and identity of the territory have a determining influence on the overall experience of the tourist, which highlights their singularities and endogenous resources.

The relationship found between the emotional image of the territory and the overall experience (H3) agrees with the results of San Martín and Del Bosque (2008). The present findings also agree with Prayag et al. (2017) although, in the studies cited, the researchers also included the tourist's satisfaction in their relationships.

Regarding H4, the results obtained agree with the results of Kim et al. (2012) regarding the positive influence that an overall experience in the destination has on tourist loyalty. Therefore, an overall experience in a territory with identity in the context of quality local gastronomic products (such as EVOO) and with a positive emotional image, is beneficial for tourist loyalty. These results confirm the general consensus in the literature about the influence of the overall experience on tourist loyalty (Chi and Qu, 2008).

The main results of this model reveal that both food tourists' perceptions of territory identity (17.7%) and emotional image (17.5%) are significant predictors for the overall tourist experience (35, 3%). For its part, the identity of the territory is in turn significantly positive on the emotional image of it (0.57%). Finally, the overall experience plays an important role (42.8%) as a result of the explanation of tourist loyalty, as it is seen as a factor that fosters tourist loyalty both to the territory and to the experiences linked to EVOO.

In this way, the results achieved emphasize the importance of the variables analysed as key elements to be considered by tourism managers when designing plans to promote the territory and the marketing of unique quality foods (Murgado, 2013; López-Guzmán and Sánchez-Cañizares, 2012; Horng and Tsai, 2010). In the context of the destination studied, these activities can be aimed at promoting olive oil tourism by linking it to a sustainable territory with its own identity.

This study sought to highlight the importance of the tourists' experiences in a sustainable destination, as a key factor in their intentions of loyalty for the future. The work is contextualized in territories with their own identity, based on their local quality food as a differential factor, such as extra virgin olive oil. These are cultural and gastronomic activities, which often take place outdoors in contact with nature, and which can contribute to the tourist development of these types of rural areas.

The results provide empirical support for the proposed relationships between the identity of the territory, its emotional image, the overall experience of the tourist and their loyalty. In this way, the research hypotheses are valid and the findings coincide with previous research (Baloglu and Brinberg, 1997; Chi and Qu, 2008; Pike and Ryan, 2004; Prayag et al., 2017; Llodrà-Riera et al., 2015; San Martín and Del Bosque, 2008; Kim et al., 2012). Once validated the theoretical model proposed through analysis of the data obtained in the survey, it can be said that there is a positive relationship between the identity of the territory and the emotional image of it. The positive relationship between both dimensions and the overall experience is also verified. Finally, the positive relationship between the overall experience of tourists and their willingness to return or recommend the destination and/or experiences based on the EVOO was verified. The results achieved confirm that the identity of the destination and its emotional image play a fundamental role for gastronomic tourists.

In light of the results obtained, the combination of the identity and the emotional image of the territory offer a positive overall experience to the olive oil tourist during their trip. This overall experience also positively influences the tourist's intention of recommending or revisiting the destination in the future. These data suggest an opportunity to boost tourism development of rural settings that can rely on a unique gastronomic product, since it can represent a key factor of its territorial identity helping to differentiate it from other territories.

## 6 Conclusions

The main theoretical contribution of this work is the key role played by the tourist experience in their future intentions in terms of returning or recommending the experience lived in a territorial environment with an identity and quality food. On the one hand, this study contributes to expanding the literature on olive oil tourism. On the other, the study advances the knowledge of the key predictors of tourism development in this context.

In addition, the strategies undertaken by tourism managers can be aimed at promoting the importance and differentiation of quality local foods, as is the case of tourism based on olive oil. Tourism managers can use this information to design unique tourism experiences based on local food, supported by a territory with an identity. This tourism development must be implemented in accordance with the improvement of the quality of life of residents and tourists, together with the preservation of the environmental heritage in a sustainable manner (Ellis et al., 2018; Hall et al., 2004; Everett and Aitchison, 2008). Quality local products can find a new marketing channel with their sales to tourists during the visit to the destination and when these return to their places of origin. The producers of these foods, in this case the extra virgin olive oil, can rely on the quality brand represented by the Protected Designation of Origin Monterrubio which is directly sold to tourists. In this way an interesting link would be formed between the territory, the experiences and the food with quality brands that are produced there.

The limitation of this study lies in its specific geographical context and in the analysis of a specific tourist activity based on olive oil. Future research can replicate this study in other places with identity and with different foods of recognized quality, in order to test the scope of the results obtained. This could improve the development plans of tourist destinations based on quality food, guaranteeing their success by including the component of the tourist experience. A longitudinal study could be the objective of another study to identify changes in the profile/behavior of this tourist.

## Declarations

### Author contribution statement

José Antonio Folgado-Fernández: Conceived and designed the experiments; Performed the experiments; Analyzed and interpreted the data; Contributed reagents, materials, analysis tools or data; Wrote the paper.

Ana María Campón-Cerro: Conceived and designed the experiments; Contributed reagents, materials, analysis tools or data.

José Manuel Hernández Mogollón: Analyzed and interpreted the data; Contributed reagents, materials, analysis tools or data.

### Funding statement

This work was supported by the European Regional Development Fund, European Union, and the Consejería de Economía e Infraestructuras de la Junta de Extremadura (Spain), in the context of the Project ´AOVETUR_EXTREM. A proposal for a model to the sustainable integral development of rural areas in Extremadura based on the binomial Extra Virgin Olive Oil and Tourism (Ref. IB16104).

### Competing interest statement

The authors declare no conflict of interest.

### Additional information

No additional information is available for this paper.

## Footnotes

1 Based on information obtained from the Monterrubio PDO in http://aceitemonterrubiodop.com.
